# Tracing the evolving dynamics and research hotspots of microbiota and immune microenvironment from the past to the new era

**DOI:** 10.1128/spectrum.00135-23

**Published:** 2023-09-28

**Authors:** Runzhi Huang, Yuntao Yao, Xirui Tong, Lei Wang, Weijin Qian, Jianyu Lu, Wei Zhang, Yifan Liu, Siqiao Wang, Shuyuan Xian, Yushu Zhu, Jie Huang, Xinya Guo, Minyi Gu, Hanlin Lv, Wenshuai Bi, Chenwei Meng, Zhengyan Chang, Jie Zhang, Dayuan Xu, Shizhao Ji

**Affiliations:** 1 Department of Burn Surgery, First Affiliated Hospital of Naval Medical University, and Research Unit of Key Techniques for Treatment of Burns and Combined Burns and Trauma Injury, Chinese Academy of Medical Sciences, Shanghai, China; 2 Shanghai Jiao Tong University School of Medicine, Shanghai Jiao Tong University, Shanghai, China; 3 Beijing Genomics Institute (BGI), Shenzhen, China; 4 Tongji University School of Medicine, Tongji University, Shanghai, China; 5 Department of Pathology, Shanghai Tenth People’s Hospital, Tongji University School of Medicine, Shanghai, China; 6 Key Laboratory of Spine and Spinal Cord Injury Repair and Regeneration of Ministry of Education, Orthopaedic Department of Tongji Hospital, School of Medicine, Tongji University, Shanghai, China; Tainan Hospital, Tainan, Taiwan

**Keywords:** gut microbiota, immune microenvironment, bibliometric analysis, cancer, immunotherapy, inflammation, acute injury, metabolic diseases

## Abstract

Gut microbiota can regulate many physiological processes within gastrointestinal tract and other distal sites. Dysbiosis may not only influence chronic diseases like the inflammatory bowel disease (IBD), metabolic disease, tumor and its therapeutic efficacy, but also deteriorate acute injuries. This article aims to review the documents in this field and summarize the research hotspots as well as developing processes. Gut microbiota and immune microenvironment-related documents from 1976 to 2022 were obtained from the Web of Science Core Collection database. Bibliometrics was used to assess the core authors and journals, most contributive countries and affiliations together with hotspots in this field and keyword co-occurrence analysis. Data were visualized to help comprehension. Nine hundred and twelve documents about gut microbiota and immune microenvironment were retrieved, and the annual publications increased gradually. The most productive author, country, and affiliation were “Zitvogel L,” USA and “UNIV TEXAS MD ANDERSON CANC CTR,” respectively. FRONTIERS IN IMMUNOLOGY, CANCERS, and INTERNATIONAL JOURNAL OF MOLECULAR SCIENCE were the periodicals with most publications. Keyword co-occurrence analysis identified three clusters, including gut microbiota, inflammation, and IBD. Combined with the visualized analysis of documents and keyword co-occurrence as well as literature reading, we recognized three key topics of gut microbiota: cancer and therapy; immunity, inflammation and IBD; acute injuries and metabolic diseases. This article revealed researches on gut microbiota and immune microenvironment were growing. More attention should be given to the latest hotspots like gut microbiota, inflammation, IBD, cancer and immunotherapy, acute traumas, and metabolic diseases.

**IMPORTANCE:**

Gut microbiota can regulate many physiological processes within gastrointestinal tract and other distal sites. Dysbiosis may not only influence chronic diseases like inflammatory bowel disease (IBD), metabolic disease, tumor and its therapeutic efficacy, but also deteriorate acute injuries. While the application of bibliometrics in the field of gut microbiota and immune microenvironment still remains blank, which focused more on the regulation of the gut microbiota on the immune microenvironment of different kinds of diseases. Here, we intended to review and summarize the presented documents in gut microbiota and immune microenvironment field by bibliometrics. And we revealed researches on gut microbiota and immune microenvironment were growing. More attention should be given to the latest hotspots like gut microbiota, inflammation, IBD, cancer and immunotherapy, acute traumas, and metabolic diseases.

## INTRODUCTION

Gastrointestinal tract (GIT) of human is inhabited by complex communities of microorganisms, including bacteria, yeast, viruses, archaea, and protozoa, commonly referred to as the gut microbiota. Millions of years of co-evolution between the host and gut microbiota have led to a symbiotic relationship in which the host provides nourishing and hospitable surroundings to gut microbiota, and in the meanwhile the gut microbiota contributes to many aspects of host physiological functions (1, 2). For instance, in the gut, it modulates nutrient absorption, promotes the intestinal epithelial barrier integrity, immune homeostasis, and protection against pathogen colonization (3, 4). Moreover, the gut microbiota also influences the whole host system such as regulation of metabolism, inflammation, and immunity (5). Nonetheless, because of the constant exposure to the foreign antigens and gut microbiota with changeful compositions, homeostasis of GIT immune system is always threatened. Disruption of immune homeostasis can lead to intestinal inflammation and systemic diseases including cancers (6, 7).

The bacterial component takes up the majority of the gut microbiota, and recent studies have found that many specific kinds of bacteria and their metabolites will influence the immune microenvironment and promote tumorigenesis. For example, *Fusobacterium nucleatum* was proposed to be correlated to the initiation, progression, and prognosis of colorectal cancer and it may also influence the therapeutic effect (8 – 13). Additionally, metabolites like short-chain fatty acids (SCFAs) are evidenced to regulate many immune cells to influence the immune microenvironment, and secondary bile acids may promote the liver cancer (14). Furthermore, with the increasing knowledge of immunotherapy and gut microbiota, the variety and composition of gut microbiota has been evidenced to partly determine the immunotherapeutic efficacy, and thus, strategies like fecal microbiota transplantation (FMT) and combination of probiotics with immune checkpoint inhibitors (ICIs) are now being studied (10, 15 – 21). Moreover, gut microbiota also plays a significant role in the disease progression, prognosis, and recovery of various acute injuries including acute central nervous system (CNS) injuries and burn injuries. For example, these acute injuries will probably lead to compromised gut barrier and increasing leakiness, causing dysbiosis and bacterial translocation in a short period of time (22 – 24). Dysbiosis and gut barrier disruption have been found to impact the acute injuries through different mechanisms (22, 23). Therefore, figuring out the explicit relationship and mechanisms are very important for the treatment and for providing new treatment options for patients.

There is an increasing number of articles being published every year on gut microbiota and immune microenvironment. Hence, it is imperative for scholars to keep an appreciable and constant update of the latest hotspots. As a quantitative analytic approach, bibliometric analysis can depict the knowledge structure and development trends in a specific research field based on science mapping (25). With the application of mathematical statistics, it enables statistical analysis of humongous volumes of information to obtain a comprehensive understanding of a certain field, including the identification of influential journals, authors, affiliations, and countries, detecting trending topics and hotspots over time. Bibliometric analysis has gained huge popularity in multidisciplinary fields recently. The popularity of bibliometric analysis can be attributed to the advancement, availability, and accessibility of bibliometric software, including Citespace, VOSviewer, R-bibliometrix, and scientific databases such as Web of Science (WOS). Bibliometrics has also been applied in research with regard to gut microbiota and various diseases, which all showed great impact (26 – 30). Among them, a bibliometric analysis showed that gut microbiota and host immune response remain research hotspots (26). However, their work mostly focused on the relationship between gut microbiota and innate and adaptive immunity, especially the T cells. While the application of bibliometrics in the field of gut microbiota and immune microenvironment still remains blank, which focused more on the regulation of the gut microbiota on the immune microenvironment of different kinds of diseases. Here, we intended to review and summarize the presented documents in gut microbiota and immune microenvironment field by bibliometrics, which can help researchers quickly catch the whole general framework of this field and obtain the several researching hotspots in recent years and future.

## MATERIALS AND METHODS

### Data sources and retrieval strategies

WOS Core Collection database was used as the data source because WOS is a high-quality digital literature resource database, covering an extensive range of publications in multiple fields and is widely acknowledged as the most appropriate database for bibliometrics analysis (31 – 33).

Bibliometric analysis in gut microbiota and immune microenvironment was performed using the WOS core database as of 5 July 2022. The retrieval strategy was demonstrated as follows. The subject words contained gut, microbiota, and immune microenvironment, together with their other synonyms. The detailed retrieval formula was {[(TS = inflammatory microenvironment) OR (TS = inflammation microenvironment) OR (TS = immune microenvironment)] AND [(TS = gut) OR (TS = intestin*) OR (TS = gastrointestin*) OR (TS = gastro-intestin*)] AND [(TS = microbiot*) OR (TS = microbiome*) OR (TS = flora) OR (TS = microflora) OR (TS = bacteria) OR (TS = prebiotic) OR (TS = probiotic)]}. Finally, a total of 912 documents were retrieved and downloaded in a text from WOS core database for further bibliometric analysis.

### Methodology and data analysis processes

Bibliometrics is a method widely applied in literature analysis as a quantitative method for reviewing and extracting key information from the published literature (34). On the basis of analyzing authors, journals, institutions, countries and keywords, and then the performance analysis, evolution and hotspots of a field can be generally acquired (35). Bibliometrix is an open-source tool which can be used with the help of R language and R studio, plus biblioshiny. Biblioshiny is a web-based operator, which is developed in R version 4.2.0 (Institute for Statistics and Mathematics, Vienna, Austria; www.r-project.org), and could provide a web operating interface for bibliometric analysis (36). After uploading the documents data of the foregoing downloaded text into biblioshiny, it can help provide a quantitative assessment, visualizing sources, authors, and documents’ analysis for the large volumes of scientific data (37).

Co-citation and word co-occurrence analyses are both commonly used methods in bibliometrics. If two articles are cited by other articles in the meantime, it is called co-citation. Similarly, word co-occurrence analysis is applied to discover words that usually appeared together in a same article. Both of them can assist researchers to make sense of the internal relationship and degree of closeness about the researching hotspots of this field better.

Moreover, by using modern computer technology, visualization can be achieved by graphs to present a more intuitional view, which largely facilitates data interpretation. VOSviewer and Citespace are the two most used software for picturing, which have their own advantages. VOSviewer is good at providing network visualization and density visualization, helping the keywords, and co-occurrence analysis, while Citespace can provide precise timeline view, clearly presents the keyword dynamics and developing processes in different time (38, 39).The two computational tools were also used in our bibliometrics study.

H-indices are utilized to evaluate a scholar’s scientific influence and outputs in a concise and useful manner. It means that for each scholar, h of his documents have at least h citations, while his other documents have less than h (40). It provides an estimation of the broad scientific impact of a researcher in an evenhanded way.

## RESULTS

**Fig 1:**
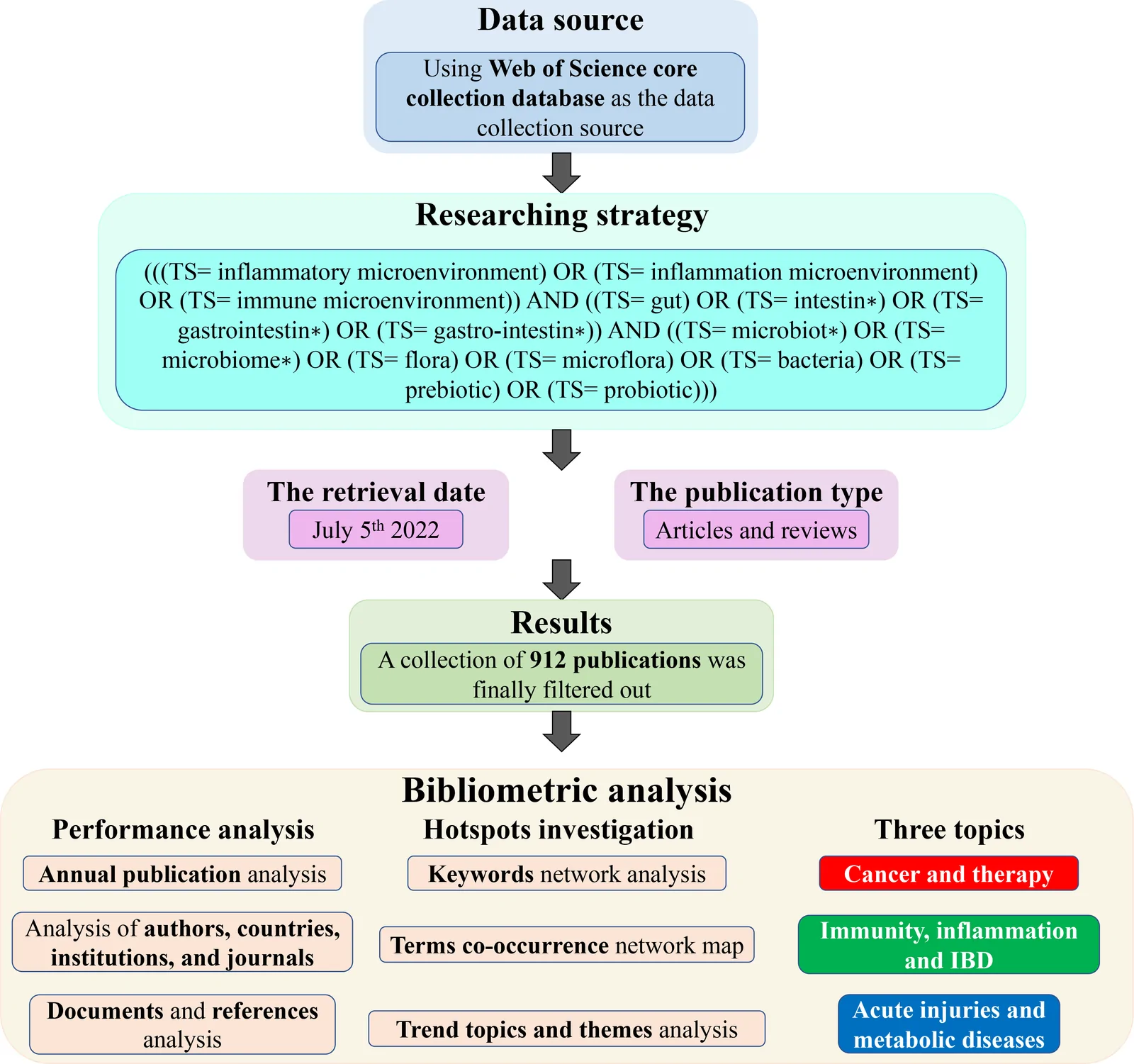
The schematic flowchart of our study. We demonstrated the data source, process of researching, and bibliometric analysis in the picture.

A total of 912 documents collected from 1976 to 2022 for the analysis were written by 4,440 authors, published in 394 journals with an average citation of 54.08 per document, and 44,323 references were cited by these documents altogether. In order to make our study more readily understood, we portrayed a schematic picture, as shown in Fig. 1, which vividly demonstrated the process of our study.

### Analysis of annual publications

**Fig 2:**
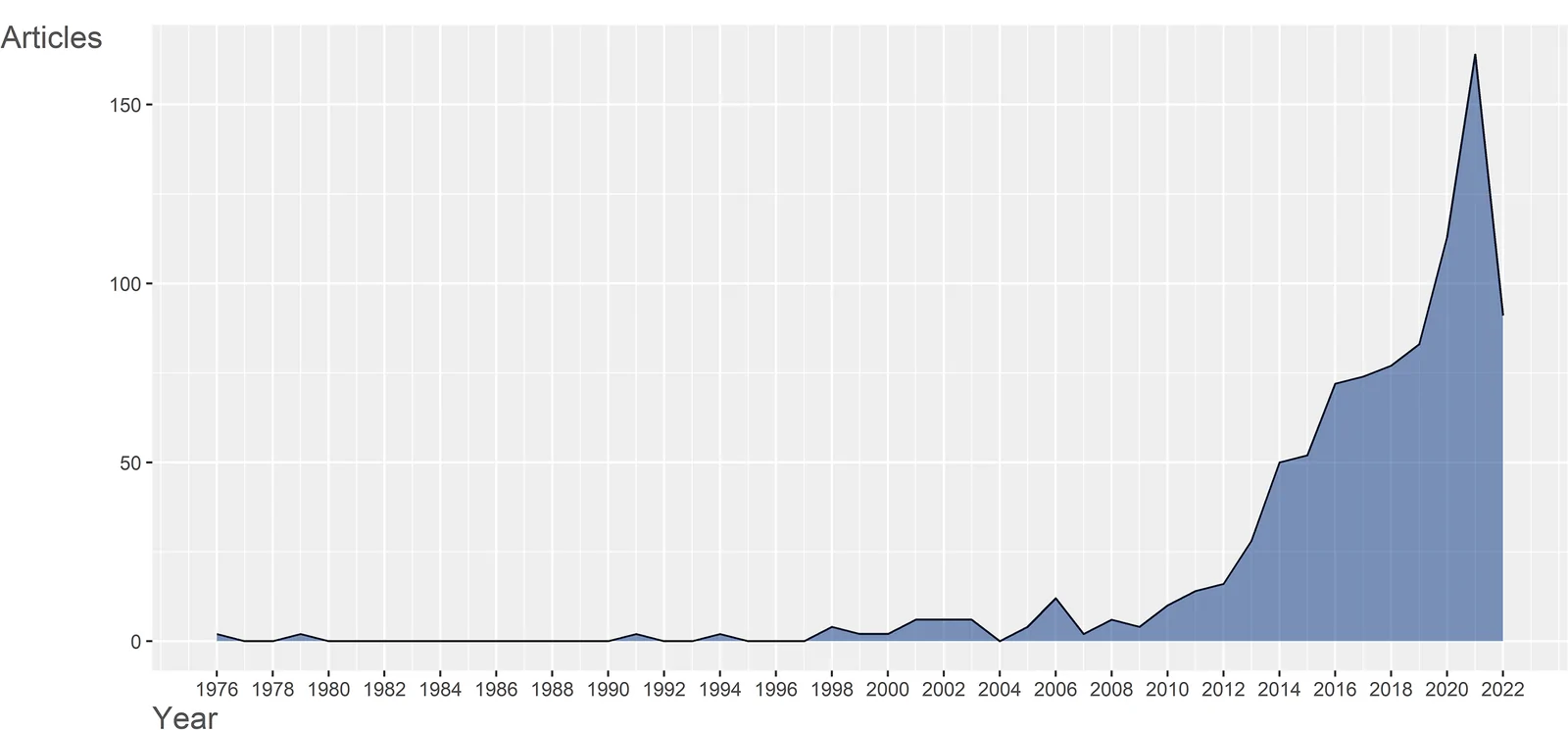
Annual publications in gut microbiota and immune microenvironment field from 1976 to 2022. The publications before 2010 were sporadic and always less than 10, while after 2010, the yearly publications sustained rapid growth, reflecting the rapid development of the gut microbiota and immune microenvironment field in recent years.

The number of annually published articles can in general reflect the attention paid by researchers in a specific researching field. As pictured in Fig. 2, yearly publications in gut microbiota and immune microenvironment field were sporadic and almost always less than 10 publications a year before 2010, indicating the immaturity of this field. However, after 2010, the publications number began to sustain rapid growth, and reached a peak of more than 150 publications outputs in 2021 (2022 outputs may continuously exceed 2021 with more than 90 articles published in the first half year). Despite the fact that there were great fluctuations in average article citations per year, as showed in Fig. S1, it could also reflect the rapid development in recent years.

Recent growth of publications in this field was greatly contributed by the revolution of the advances in cancer immunotherapy and new investigative methods. On one hand, cancer immunotherapy has been really high-profile recently because it proved to be really successful in fighting against a variety of malignancies (41 – 46) and, thus, a lot of researches were focusing on the complex relationship between the cancers as well as the immunotherapy resistance and gut microbiota components. On the other hand, with the advent of investigative methods like 16S RNA sequencing and the next-generation sequencing, they greatly facilitated researchers to know the composition of gut microbiota and to align the whole genomes of every single bacterium in an unbiased way. Also, it is much more efficient to acquire the profiling of complicated microbiome species in diverse environments and to analyze the different changes of the bacterial composition over time. In addition, improved bacterial culturing techniques have made it possible to isolate the bacteria species that were once thought unculturable (47 – 49). Furthermore, the increasing uses of germ-free mice have enabled studies of detailed mechanisms of interaction between gut microbiota and both cancers and host immune system (50).

### Contributions of authors, countries, and institutions

**Fig 3:**
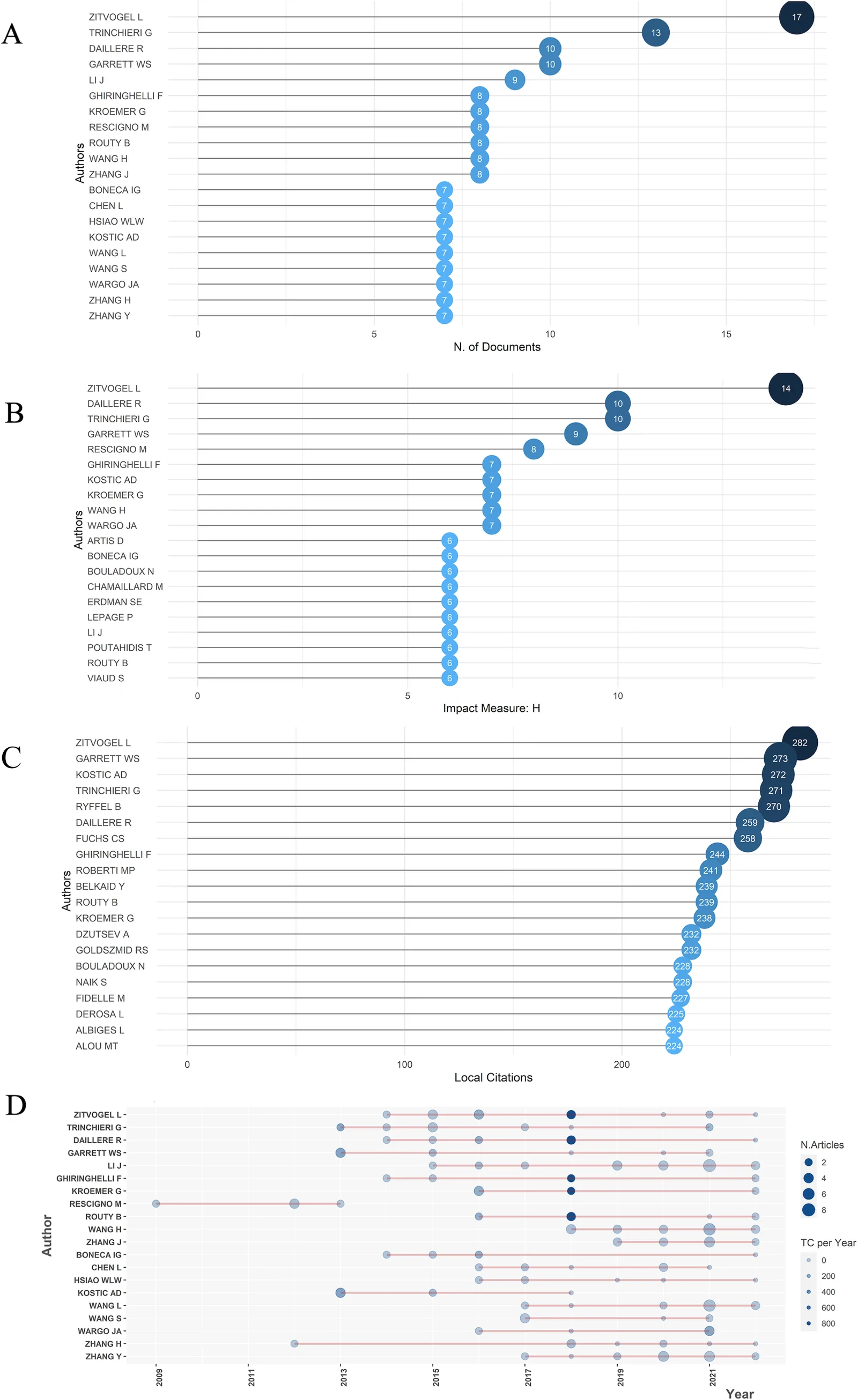
(**A**) The top 20 most relevant authors who have published most of the articles in the gut microbiota and immune microenvironment field were listed accordingly. “Zitvogel L” and “Trinchieri G” were the top 2 authors, publishing 17 and 13 documents. respectively. (**B**) The top 20 authors’ local impact measured by h-index, which means that an author has published h articles and each of them has been cited for at least h times. It was noteworthy that “Zitvogel L” had the highest h-index of 14. (**C**) The top 20 most local cited authors, and local citation of a document meant that it was cited by any other document in our retrieval collection. The most local cited author was also “Zitvogel L” with 282 local citations, and other 19 authors were all locally cited for more than 200 times. (**D**) The top 20 authors’ production over time; the length of line means the author’s researching timeline; the size of the nodes reflects the number of publications; and the color density is proportional to total citations per year. It was worth pointing out that 18 of 20 authors were still active in the recent 2 y, again emphasizing that this was a newly emerging field.

Up to 4,420 authors have published their studies in this field by the retrieved date (5 July 2022). Analyzing the most productive and influential authors can help researchers know part of most important investigation fields. The frequency distribution of scientific productivity was put in Fig S2A, which indicated that most authors in this area published articles less than five. While in Fig. 3A, the top 20 authors who have published the most articles were listed accordingly with a minimum of 7. “Zitvogel L” and “Trinchieri G” published 17 and 13 documents and took the first and second place, respectively, surpassing other authors. H-index means that author has published h articles and each of them has been cited for at least h times (51), which can measure the quantity and the quality of the authors’ articles in a meantime. The h-index range of the top 20 authors was from 6 to 14, and the author with highest h-index was also “Zitvogel L” (Fig. 3B). Furthermore, local citation of a document meant that it was cited by any other document in our retrieval collection, and documents, as well as authors, with a high number of local citations suggesting their prominent impacts in this field. And the most local cited author was also “Zitvogel L” with 282 local citations, and other 19 authors were all locally cited for more than 200 times (Fig. 3C). In Fig. 3D, the horizontal line meant the timespan that an author was doing the related researches, while the bigger size and darker color shades of the nodes, respectively, represented the larger number and higher total citations per year. It was worth pointing out that 18 of 20 authors were still active in the recent 2 years, again emphasizing that this was a newly emerging field. As Fig. 3 presented, “Zitvogel L” published 17 articles with 282 local citations, 5,710 total citations, and an h-index of 14, dominating all these statistical rankings, indicating that “Zitvogel L” was the most productive and influential author. His researches mostly focused on the relationship between immunology and cancer, and his most influential study in this field put forward that altered gut microbiota composition would lead to immune checkpoint inhibitors primary resistance, which has been cited for more than two thousand times.

**Fig 4:**
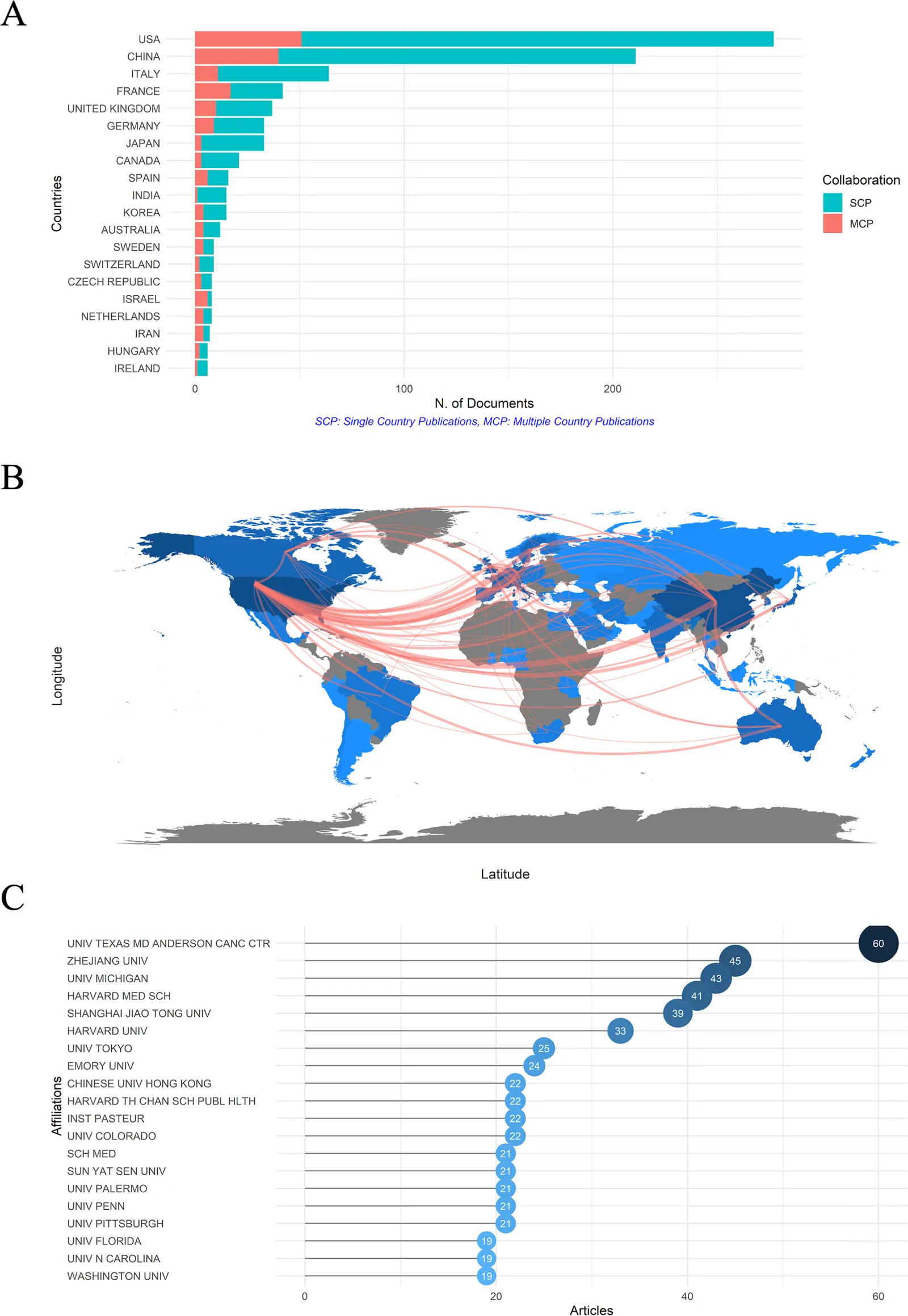
(**A**) The top 20 productive countries which were measured by the number of documents written by corresponding author’s countries. SCP means that the investigation was finished by only one country, while MCP means that the researches were conducted by multiple countries. High MCP and high MCP ratio suggested that the researches of the country were more likely to cooperate with others, possibly implying that the development of this field would be more advanced. (**B**) Visualization of collaboration among countries by a network map; the line number means the cooperation times between countries, and the color density is proportional to the total publications. (**C**) The top 20 affiliations with the most publication outputs. American and Chinese affiliations are the two most prolific affiliations in the gut microbiota and immune microenvironment field. SCP, single country publication; MCP, multiple countries publication.

To determine the most productive and influential countries more directly, the bibliometric analysis of the several leading countries was listed in Table 1. Countries were ranked by the total citations, which combined both the quantity (number of records) and the quality (citations per item). The total citations of each country are displayed and can be intuitively seen in Fig. S2BC. The darker the color, the higher the total citations. The top 3 countries in sequence were USA, France, and China. The USA published 277 records and got 73.90 citations per item, followed by France with 42 records and 167.07 citations per item, while China published 211 records but only 27.76 citations for each item. SCP (single country publication)refers to research completed solely by scientists from one country. In contrast, MCP (multiple countries publication) indicates research conducted collaboratively by scientists from multiple countries, offering a partial reflection of the level of cooperation between different countries. The USA, China, and France were the top 3 countries with most MCPs (Table 1; Fig. 4A), indicating that they were the countries most willing to cooperate, which could also be directly seen in the collaborative network map, and high MCP and high MCP ratio possibly implied that the development of this field would be more advanced (Fig. 4B). Dense lines between the two countries indicated high cooperation times. The USA had the greatest amount of cooperation with other countries, especially with China and Western Europe. Additionally, the darker the color of the country, the more the publications; so, in this field, most studies were done by North America, China, and Western European scientists.

**TABLE 1:** Top 10 most contributing countries

Rank	Country	Total citations	Records	Citations per item	MCP^*a*^
**1**	USA	20,469	277	73.90	51
**2**	France	7,017	42	167.07	17
**3**	China	5,857	211	27.76	40
**4**	UK	3,321	37	89.76	10
**5**	Japan	2,475	33	75.00	3
**6**	Italy	2,472	64	38.63	11
**7**	Germany	828	33	25.09	9
**8**	Spain	572	16	35.75	6
**9**	Canada	466	21	22.19	3
**10**	Portugal	412	4	103.00	3

^*a*^ MCP, multiple countries publication.

As presented in Fig. 4C, the top 20 most prolific affiliations were listed, and “UNIV TEXAS MD ANDERSON CANC CTR” topped the rank with a total of 60 publications. Together with the third, fourth, sixth, eighth, and tenth affiliations in the figure, there were six American affiliations in the top 10. While Chinese affiliations are also very productive with three world-famous universities in the top 10, and that is “ZHEJIANG UNIV,” “SHANGHAI JIAO TONG UNIV,” “CHINESE UNIV HONG KONG.” American and Chinese affiliations are the two most prolific affiliations in the gut microbiota and immune microenvironment field.

### Journal analysis

**Fig 5:**
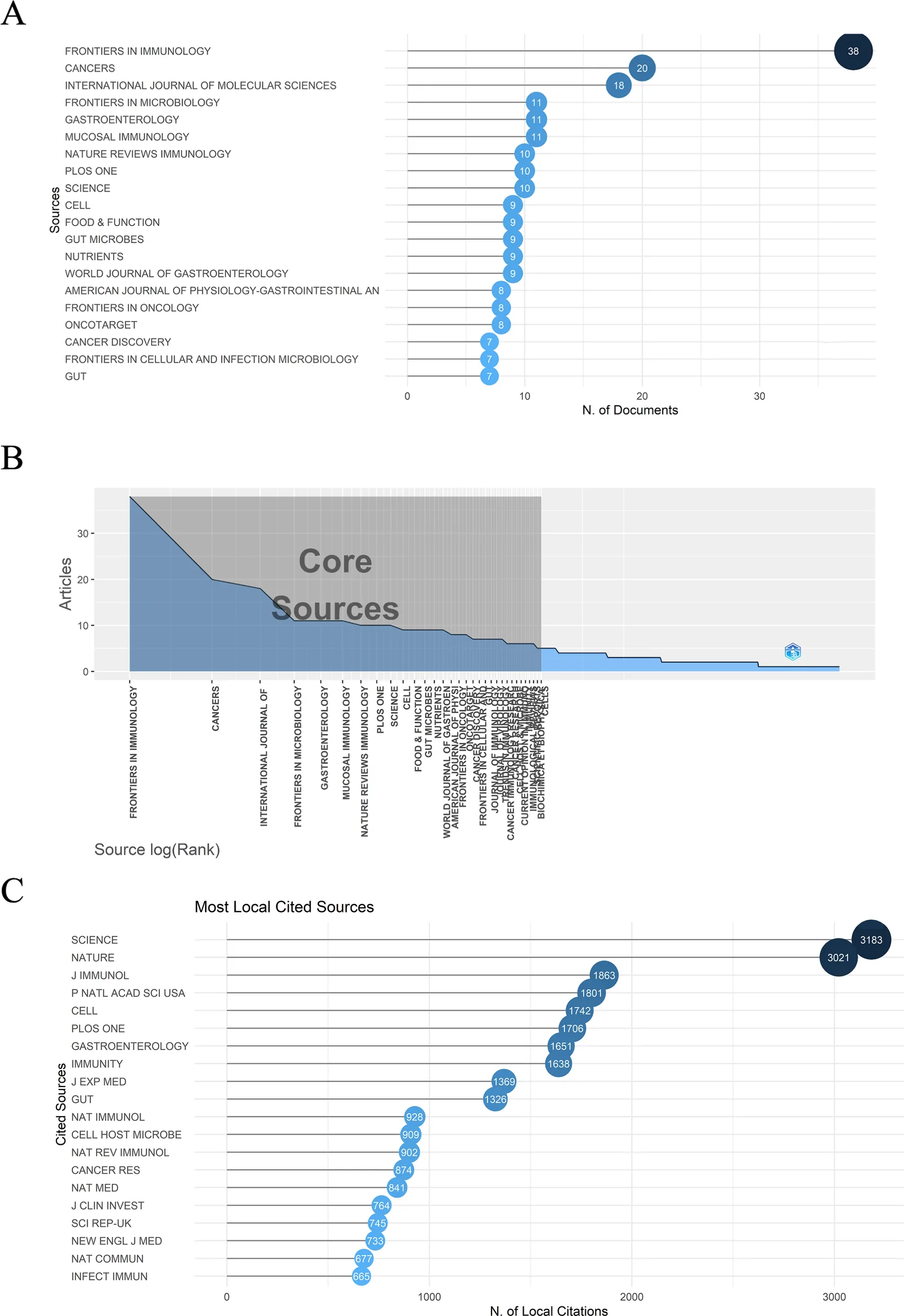
(**A**) The top 20 most relevant journals. (**B**) Journals’ order of arrangement corresponded to their publication outputs. All of the 20 most relevant journals were among the core sources, meaning that they were all pronounced in this area. (**C**) The top 20 most local cited journals, with Science and Nature dominated in this field.

Altogether, 394 journals have published articles in the gut microbiota and immune microenvironment field. Fig. 5A demonstrated the most relevant 20 journals with a minimum of 7 published articles. Overall publications of the 20 journals were 229, accounting for 25.11% of all publications. In addition, according to the Branford’s law, which helped evaluate the exponentially diminishing returns of searching references in scientific journals, all of the 20 journals were among the core sources, meaning that they were all pronounced in this area. Placing emphasis on documents from these core journals would be surely conducive to catch the latest trend efficiently. The result could be visualized in Fig. 5B. Journals with the top 20 local impact measured by h-index and the several journals with the highest publication cumulative occurrences could be viewed in Fig. S3A and S3B, respectively. In Table 2, the detailed information of the top 20 relevant journals was listed, including the number of publication articles, proportion, impact factor, and quartile in category. The predominant subject belonged to gastrointestinal, cancer immunology, and microbiology. FRONTIERS IN IMMUNOLOGY, CANCERS, and INTERNATIONAL JOURNAL OF MOLECULAR SCIENCE were the top 3 periodicals with most publication, which transcended other journals a lot. Notably, 13 of the journals were in the Q1 quartile and 6 of them had an impact factor (IF) more than 30 in 2021, reflecting the high-quality of these journals. Fig. 5C shows the most local cited journals were the two top journals in the scientific field: SCIENCE and NATURE. Both were cited for more than three thousand times.

**TABLE 2:** The top 10 highly-productive journals in gut flora and immune microenvironment field

Rank	Journal	Number of publications	Proportion, %	IF^*a*^ [2021]	Quartile in category [2021]
1	FRONTIERS IN IMMUNOLOGY	38	4.17	8.786	Q1
2	CANCERS	20	2.20	6.575	Q1
3	INTERNATIONAL JOUNRAL OF MOLECULAR SCIENCE	18	1.97	6.208	Q2
4	FRONTIERS IN MICROBIOLOGY	11	1.21	6.064	Q1
5	GASTROENTEROLOGY	11	1.21	33.883	Q1
6	MUCOSAL IMMUNOLOGY	11	1.21	8.701	Q1
7	NATURE REVIRWS IMMUNOLOGY	10	1.10	108.555	Q1
8	PLOS ONE	10	1.10	3.752	Q2
9	SCIENCE	10	1.10	63.714	Q1
10	CELL	9	0.99	66.85	Q1
11	FOOD & FUNCTION	9	0.99	6.317	Q1
12	GUT MICROBES	9	0.99	9.434	Q1
13	NUTRIENTS	9	0.99	6.706	Q1
14	WORLD JOURNAL OF GASTROENTEROLOGY	9	0.99	5.374	Q2
15	AMERICAN JOURNAL OF PHYSIOLOGY GASTROINTESTINAL AND LIVER PHYSIOLOGY	8	0.88	4.871	Q2
16	FRONTIERS IN ONCOLOGY	8	0.88	5.738	Q2
17	ONCOTARGET	8	0.88	5.168	Q2
18	CANCER DISVCOVERY	7	0.77	38.272	Q1
19	FRONTIERS IN CELLULAR AND INFECTION MICROBIOLOGY	7	0.77	6.073	Q2
20	GUT	7	0.77	31.793	Q1

^*a*^ IF, impact factor.

### Documents and references analysis

**Fig 6:**
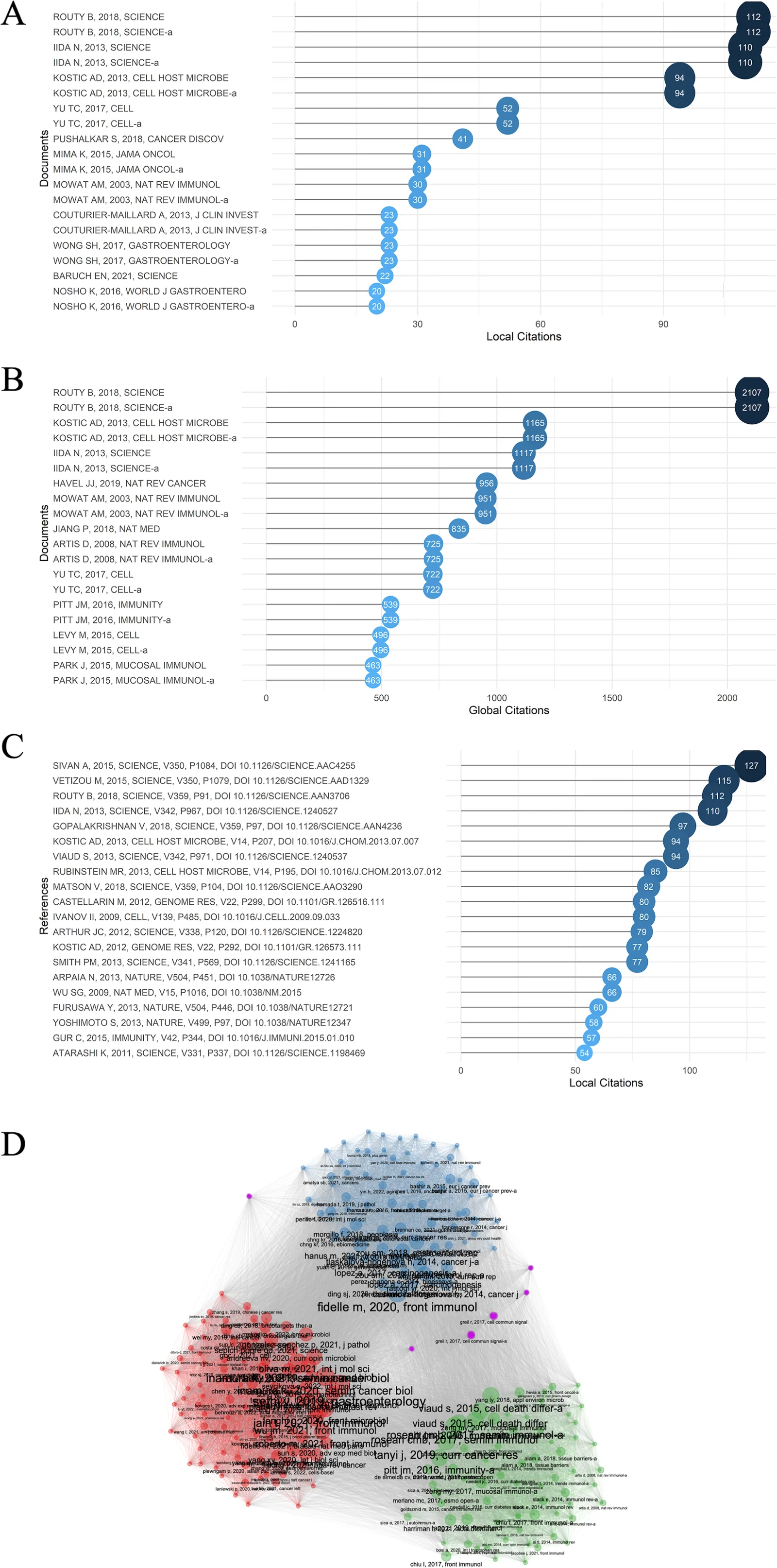
(**A**) The top 20 most local cited documents. Documents meant the publications that we have downloaded from Web of Science core database with our retrieval text. (**B**) The top 20 most global cited documents. (**C**) The top 20 most local cited references. The references were the references of the documents we have previously retrieved, so that references with high local citations were more likely to have great influences inside this field. (**D**) Documents clusters. The four clusters were in red, blue, green, and purple.

It is acknowledged that the number of citations can not only measure the influence and importance of a certain document, but also the recognition within its scientific field (52). Hence, identifying the documents with high citations could help recognize the topics that were important and received the most attention in the past. Local citation meant that the document was cited by the other documents in our retrieval collection, indicating their influence in this particular field, whereas global citation of a document meant that it was cited by any other document in the Web of Science database, suggesting their overall influences. The top 20 local and global cited documents were displayed in Fig. 6A and B.

Intersecting the contents of the paper in the two figures, it was found that the relationship between the gut microbiota and tumor immune microenvironment, together with their influence on the cancer and immunotherapy, was probably one of the hottest topics in this field. Gut microbiome influences efficacy of PD-1-based immunotherapy against epithelial tumors (18), *Fusobacterium nucleatum potentiates intestinal tumorigenesis and modulates the tumor-immune microenvironment* (8), and Commensal bacteria control cancer response to therapy by modulating the tumor microenvironment (53) were the top 3 most cited documents locally with 112, 94, and 110 times citations, and globally 2,107, 1,165, and 1,117 times, respectively. In Fig. 6C, “reference” meant the references of the documents that we have retrieved. And if the references were cited by many of the documents we have retrieved, it probably indicated that these references had great impact in this field. Together with the 3 documents talked above, the *Commensal Bifidobacterium promotes antitumor immunity and facilitates anti-PD-L1 efficacy* (17), *Anticancer immunotherapy by CTLA-4 blockade relies on the gut microbiota* (16), and *Gut microbiome modulates response to anti-PD-1 immunotherapy in melanoma patients* (20) are the six top local cited reference. These documents were probably the cornerstones of the field, intended for figuring out the clear relationship the detailed mechanisms how gut microbiota affected the differentiation and function of immune cells in the tumor microenvironment and how gut microbiota change tumor immunosurveillance and the therapeutic efficacy of PD-1 and CTLA-4 blockades as well as chemotherapy. And specifically, some oncogenic bacteria such as *Fusobacterium nucleatum* and beneficial bacteria like several *Bifidobacterium* species were gradually recognized by epidemiological and researching analysis, while the causality needed further investigation in more situations. These foregoing influential articles paved the way for further researches in the future.

In Fig. 6D, all the documents were classified into four clusters by the keywords plus (which is author’s keywords plus the keywords added by WOS). The red, blue, and green clusters are the most recognized clusters. In the red cluster, several most cited articles were also focusing on the relationship between gut microbiota and cancer and immunotherapy to further solidate the foregoing conclusion and to promote application in patients. Experimental researches and clinical trials showed that fecal microbiota could change immune cells infiltration and gene expression to improve anti-PD-1 therapy and short-chain fatty acid concentrations were also linked between PD-L1 efficacy (54, 55). Also, more researches focused on the bacteria distal to the gut. In pancreas, several unique bacteria could drive the suppressor cells through TLR ligation, promote T cells anergy and oncogenesis (56). In blue cluster, these articles mostly paid attention to the *Fusobacterium nucleatum* effect on cancer and cancer therapy. As what has been talked above, after *Fusobacterium nucleatum potentiates intestinal tumorigenesis and modulates the tumor-immune microenvironment* (8) was published, researches on the influence and controlling of *F. nucleatum* effects were done in large quantity. *F. nucleatum* would lead to chemotherapy resistance through modulating autophagy (9). And an antibiotic called berberine could abrogate *F. nucleatum*-induced colorectal tumorigenesis effect through modulating the tumor microenvironment plus blocking the tumorigenesis-related pathways (57). In green cluster, the emphasis was put on the regulation of the immune homeostasis in the gut. For instance, Treg cells play a significant role in the suppression of the intestinal immune responses in the presence of specific T cell receptors for intestinal antigens (58)). Other cells like epithelial cells, once considered as a simple physical barrier, can recognize gut microbiota to maintain the immune homeostasis (58). Taurine, histamine, and spermine, the metabolites of gut microbiota, also can shape the intestinal interface through co-modulating NLRP6 inflammasome signaling (59).

### Research hotspots: keywords network analysis

**Fig 7:**
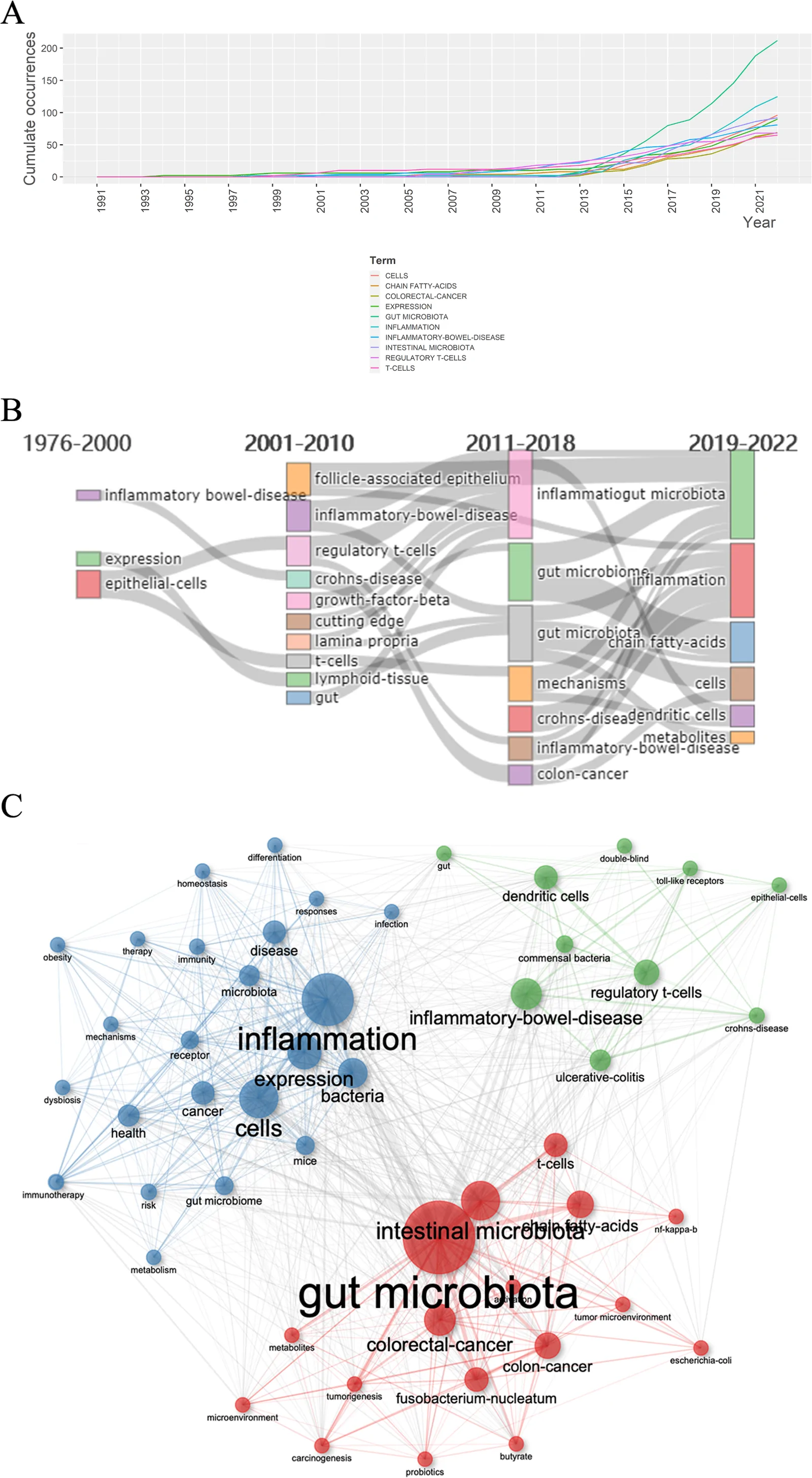
(**A**) Word growth of keywords from 1991 to 2022, and the several most frequent keywords all began to be used increasingly around 2014. (**B**) Evolution of keywords on the basis of occurring frequency at four different periods. (**C**) Keyword co-occurrence map of 50 words, and the three clusters were in red, blue, and green. Red, blue, green clusters were dominated by “gut microbiota,” “inflammation,” and “inflammatory bowel disease” accordingly.

Keywords of a certain article are highly refined, which can summarize the major topics, unfolding the most important information of the article together with the abstract. Therefore, extracting and analyzing keywords from the publications in a certain subject by bibliometrics can demonstrate major themes and then help find hotspots. Totally, 2,259 keywords plus were extracted and the top 10 most frequent keywords are presented in Table 3. Also, the picture of the top 10 most frequent keywords and keywords tree can be seen in Fig. S4. The most frequent keyword searched was “gut microbiota” with 212 times (9.38%), followed by “inflammation” with 125 times (5.53%) and “cells” with 96 times (4.25%). The fourth keyword was “intestinal microbiota” with 94 times (4.16%), which was synonymous with “gut microbiota,” and the fifth was “expression” with 90 times (3.98%). Fig 7A revealed that the several most frequent keywords all began to be used increasingly around 2014, which was accorded to the new-emergence of this period.

**TABLE 3:** Top 10 most frequent keywords plus

Rank	Keywords	Frequency, n	Percentage, %	Cumulative percentage, %
1	Gut microbiota	212	9.38	9.38
2	Inflammation	125	5.53	14.92
3	Cells	96	4.25	19.17
4	Intestinal microbiota	94	4.16	23.33
5	Expression	90	3.98	27.31
6	Inflammatory-bowel-disease	81	3.58	30.90
7	Colorectal-cancer	70	3.10	34.00
8	Chain fatty-acids	69	3.05	37.05
9	Regulatory T-cells	68	3.01	40.06
10	T-cells	66	2.92	42.98

Word dynamics could concisely exhibit the researching trend changes. In Fig. 7B, it presented the most frequently used keywords in different years. We could find that before 2011, “inflammatory-bowel-disease,” “follicle-associated epithelium,” “regulatory T cells,” and “Crohn’s-disease” were most recognized phrases, meaning that researchers were more interested in the gut epithelium structure with its immune microenvironment and their effects on inflammatory bowel diseases such as Crohn’s disease and ulcerative colitis. During 2011-2018, the important regulatory role in immune microenvironment of gut microbiome was recognized step by step. Words like “inflammation,” “gut microbiome,” “mechanisms,” and “colon cancer” were emerging at that time, indicating multiple researches were done on the relationship between gut microbiota and tumorigenesis, tumor progression especially the colorectal cancer. From 2019 to 2022, “chain fatty acids,” “cells,” “dendritic cells,” and “metabolites” were focused on and the detailed mechanisms in the metabolic and immune cells aspects were paid more attention, which might have great translational medicine meaning and clinical significance. Gut microbiota produce a large number of metabolites that will regulate host’s physiology, nutrition, and immunity (60, 61). For example, short chain fatty acids (SCFAs), such as acetate (C2), propionate (C3), and butyrate (C4), are the products of complex carbohydrates, processed by gut microbiota fermentation. They are highly produced in colon (62) and have been proved to have effects on different aspects of gut microbiome, gut barrier function, and metabolism (63). Moreover, SCFAs could regulate the immune responses through their effects on various kinds of cells that contain colonocytes, neutrophils, and T cells (6, 65, 66). Dendritic cells (DCs) are specialized APCs that bridge the innate and adaptive immune responses. The mucosa of intestine contains numerous subsets of DCs, each of which displays specific functions such as Th1-polarizing and immunoglobulin A (IgA) switching ability, and is regulated tightly by the microenvironment. Researches demonstrated that DC dysfunction might contribute to inflammatory bowel disease and celiac disease (66). Consequently, the detailed mechanism and the internal relationship between gut microbiota plus its metabolites with cancer and immunotherapy are the hottest issues.

### Terms co-occurrence network analysis

Keywords that frequently appear in a same article are placed in a same cluster, which form the keyword co-occurrence map. It could assist researchers to better make sense of the internal relationship and degree of betweenness about the researching hotspots of this field. The keyword co-occurrence network was constructed based on the top 50 frequently appearing terms and was grouped into three clusters, visualized in Fig. 7C. The nodes with the highest frequency in each cluster were “gut microbiota” (red cluster), “inflammation” (blue cluster), and “inflammatory bowel disease” (green cluster). We can analyze the common denominators about the terms in every cluster to further identify the relationship inside.

The first cluster is the red cluster, and in this cluster, words like “gut microbiota,” “chain fatty acids,” “colorectal cancer,” and “T cells” are recognized. They are mostly within the top 10 most common keywords that have been displayed above. Combined with that, the most popular hotspots in this field are likely to be the relationship between gut microbiota and cancer together with therapy, especially immunotherapy. Two major aspects are focused on most. First, interaction of gut microbiota and immune microenvironment, especially T cells and regulatory T cells. Second, the influences of the gut microbiota metabolites such as SCFAs on the immune regulation. In this cluster, “*Fusobacterium nucleatum*” is also recognized, and this specific bacteria have been proved to be more sufficient in human colonic adenomas compared with the surrounding tissue and in carcinoma patients relative to healthy persons (8). And as mentioned above, it quickly became a researching hit and following detailed researches were done in large quantity.

The second cluster is green cluster, since “inflammatory bowel diseases” is the most important phrase; it is evident that the immune influence of gut microbiota on the IBD including Crohn’s disease and ulcerative colitis is the main topic in this cluster. Also, normal regulation of gut microbiota on the immune microenvironment needs to be focused. Terms like “regulatory T cells,” “dendritic cells,” and “toll-like receptors” are the several most important immune regulating and functioning components. Besides, the cause and effect between regulation of gut immune microenvironment and IBD is one major topic in this field as well.

The third cluster is blue cluster, and “inflammation,” “expression,” “cells,” and “bacteria” are the most recognized words in this cluster, indicating that much attention was paid in the influencing factors on inflammation including different cellular and metabolic factors. Figuring out the underlying mechanisms of the phenomenon that had been found might have great translational medical significance.

In addition, the keyword conceptual structure map generated by multiple correspondence analysis (MCA) method divided the keywords into two clusters, also helping make out their relationships, which was displayed in Fig. S5A. The most cited and contributive documents in each cluster were, respectively, demonstrated in Fig. S5B and S5C. The corresponding topic dendrogram was also shown in Fig. S5D.

### Trend topics and themes analysis

**Fig 8:**
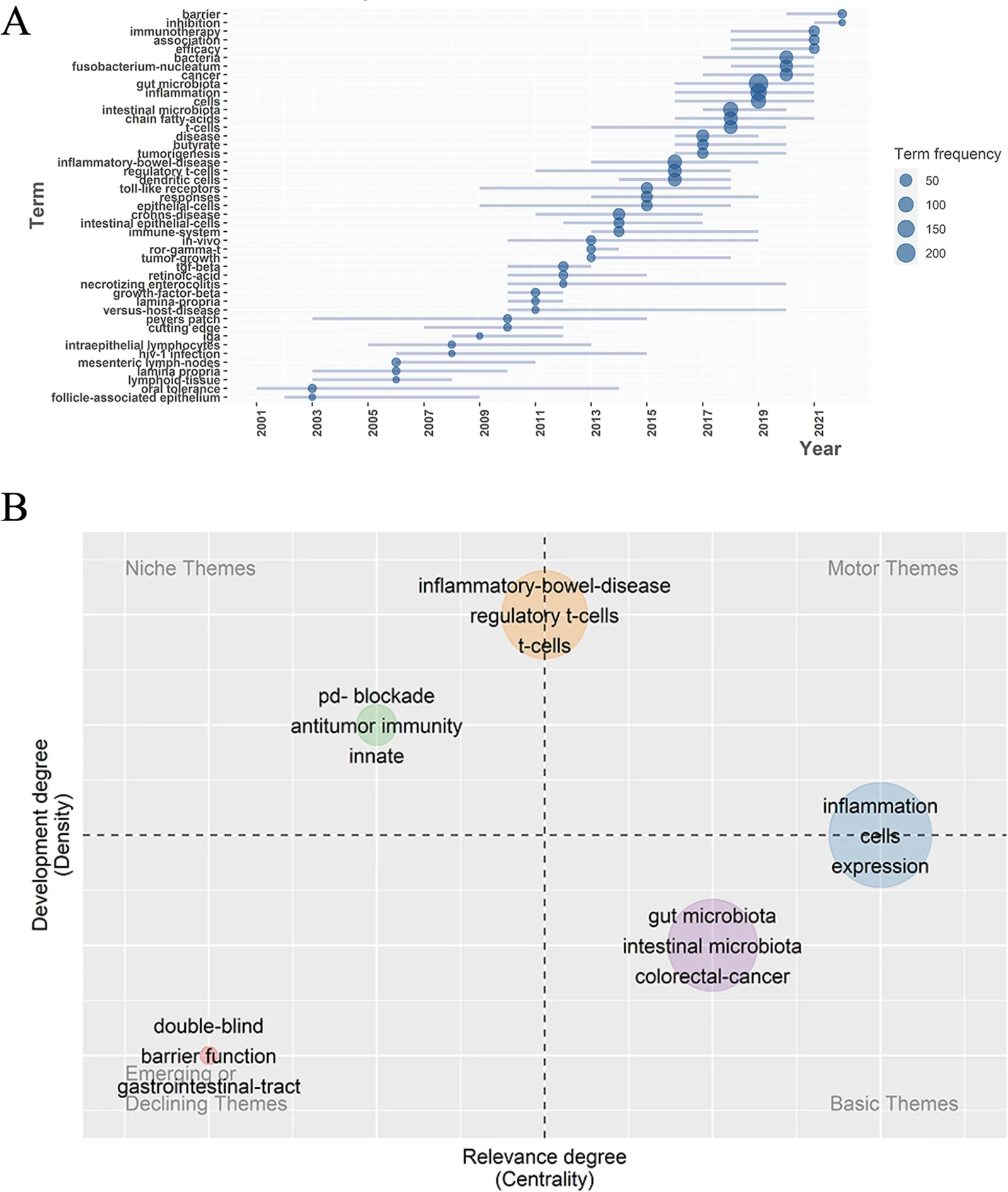
(**A**) The development of trend topics over time. (**B**) Thematic map; the X axis is centrality while Y axis is density; the map is divided into four quadrants which are motor themes, niche themes, emerging or declining themes, and basic and transversal themes, respectively.

Trend topics and themes analysis can directly show the researching potential hotspots and development of the field in a new aspect. As shown in Fig. 8A, they were the 45 major topics in gut and inflammation microenvironment field in different period of time since 2001. The several top trend topics were “barrier,” “inhibition,” “immunotherapy,” “*fusobacterium-nucleatum,*” “cancer,” “cells,” “gut microbiota,” “inflammation,” “inflammatory bowel disease,” and “chain fatty acid.” The topics like immunotherapy of immune checkpoint inhibition and cancer, inflammation and its regulatory mechanisms, and the inflammatory diseases have all been talked above. They were not only newer topics but also received the most attention, and relatively these topics would be the most promising researching directions in the near future. The barrier topic was focused on since 2019. Many researchers have found that gut microbiota had significant influence on the gut barrier, which would lead to the following diseases especially in acute injuries (67). Disrupted gut barrier will lead to bacterial translocation and following severe complications like sepsis and even multiple organ dysfunction syndrome (MODS), which greatly influence the progression of disease and prognosis of patients. As a result, the detailed mechanisms were greatly focused on. A research has found that lycium barbarum oligosaccharides, a metabolite, can increase protective bacteria such as *lactobacillus* to maintain intestinal barrier integrity (68). Disrupted gut barrier also promotes tumorigenesis. Tumorigenic bacteria like enterotoxigenic *bacteroides fragilis* might disrupt gut barrier structure, while beneficial bacterial like *A. muciniphila* could maintain the gut barrier structure, which are important for researching to treat the colorectal cancer (69).

The topics were classified and visualized as a thematic map in Fig. 8B. The X axis was “centrality,” symbolizing the relevance degree of the themes while the Y axis was “density,” representing the development degree and maturity of the field. Based on the two dimensions, four quadrants were divided and five clusters were distributed in the thematic map with nodes labeled by three keywords. The orange cluster was in the middle of the quadrant 1 and 2, meaning that the inflammatory bowel diseases were highly developed but intermediately relevant. The green cluster was in the quadrant 2 and was niche themes, representing that the immunotherapy and antitumor immunity were highly developed but less related to the field, which might be attributed to the fact that gut microbiota and its effect was just a factor of antitumor immunity besides tumor genomics and host germline genetics. The red cluster was in quadrant 3, and was emerging themes, indicating neither developed nor relevant. The blue cluster was in the quadrant 4, representing that the relationships between intestinal microbiota and colorectal cancer were basic themes. Finally, the blue cluster was the topic about inflammation and the cellular and genetic regulation mechanisms, which was in the middle of the quadrant 1 and 4, revealing the high relevance but low development of these themes.

Furthermore, to validate the results of our study, we similarly conducted the retrieval and analysis again on 29 May 2023. The results were put in the supplementary material. The annual publication was still quickly rising (Fig. S6); we could find that the results of the bibliometric analysis were all similar to the results of 2022 (Fig. S9).

## DISCUSSION

### General information

In this study, 912 documents that were related to gut microbiota and immune microenvironment between 1976 and 2022 were retrieved from the WOS core database. Bibliometric analysis and information visualization methods were conducted based on these publications, which could help us capture the development trend and hotspots in the field. The annual publications increased continuously, manifesting the popularity of the gut microbiota and immune microenvironment area in recent years, which corresponded to its great development potential and clinical significance. The rapid development was also largely facilitated by the improvement of biological techniques and promoted by the cancer immunotherapy. The most productive and influential author was “Zitvogel L,” who published 17 articles with 282 local citations, 5,710 total citations and an h-index of 14. For countries, the top 3 productive and influential were USA, France, and China, and they were also the countries that were most willing to cooperate. In terms of affiliations, in the top 10 affiliations, 6 of which were American including “UNIV TEXAS MD ANDERSON CANC CTR,” and 3 were China universities including “ZHEJIANG UNIV,” “SHANGHAI JIAO TONG UNIV.” When it comes to the journals, FRONTIERS IN IMMUNOLOGY, CANCERS, and INTERNATIONAL JOUNRAL OF MOLECULAR SCIENCE were the journals with most publication. And the most local cited journals were SCIENCE and NATURE, both cited for more than three thousand times. Co-word and clustering analysis helped identify the three research hotspots (gut microbiota, inflammatory bowel disease, and inflammation). Combined with the results of the bibliometric analysis, we want to discuss several possible hotspots as follows. In the red cluster, the influence of gut microbiota on cancer and therapy was most focused. While for the green cluster, we would discuss the role of gut microbiota in immunity and inflammatory bowel disease. At last, we would discuss the relationship between gut microbiota, inflammation, and related diseases, such as acute injuries and metabolic diseases in the blue cluster.

### The red cluster: influence of gut microbiota on cancer and therapy

Gut microbiota function would influence not only the gut mucosa, but also the systemic immune homeostasis. Investigations have found that gut microbiota is likely to not only contribute to tumorigenesis and cancer immunosurveillance, but may also augment or weaken the therapeutic effect. With our developing understanding of the complicated relationship between different host-intrinsic gut microbiota, more and more novel therapeutic strategies like fecal microbial transplantation and probiotics were intended to target gut microbiota to improve treatment efficacy in cancer (14). Consequently, making out the gut microbiota function and accurate evaluation of a patient’s microbial constitution was important for the following targeted therapeutic strategy, which would be a key aspect of future multidisciplinary and precision-medicine strategies (14, 70). Recent studies were focusing on the different effects of gut microbiota on cancers and cancer therapy, including its local and distant effects, chemotherapy, and immunotherapy.

On one hand, in terms of the local effects of gut microbiota on carcinogenesis in the gastrointestinal tract, *Fusobacterium nucleatum* was the most recognized, which was discovered to play a critical role in tumorigenesis of CRC. In CRC patients, they tended to have an elevated level of *F. nucleatum* compared normal healthy tissue. Furthermore, a higher intratumoral level of *F. nucleatum* demonstrated less overall survival than patients with lower level (12, 13). Mechanistically, *F. nucleatum* were evidenced to be related to the progression of CRC by activating β-catenin signaling, which was pro-inflammatory (8, 11). Besides, some invasive *Campylobacter* species were found to be partly responsible for tumor development by inducing a pro-inflammatory response with the help of Interleukin-18 (71, 72). Some proteobacteria, such as *Escherichia coli,* would also help CRC progress through activating senescence-associated secretory phenotype to secrete growth factors, cytokines and enzymes (14). Except the oncogenic species of bacteria, some beneficial species of gut microbiota were also recognized. *Bifidobacterium* was linked with antitumor effect. Enhanced CD8(+) T cell accumulation in the tumor immune microenvironment due to the augmented dendritic cell function helps accomplish the effect (17). In addition, *Akkermansia muciniphila* could maintain the gut barrier structure to prevent colorectal cancers (69). Also, one study found that *Streptococcus thermophilus* have the ability to secrete β-galactosidase to directly suppress tumor growth by stimulating the oxidative phosphorylation and inhibiting the glycolysis (73).

On the other hand, gut microbiota would have distant effects. For instance, by translocating through the pancreatic duct, some species of gut microbiota can inhabit in the pancreas. Some commensal gut microbiota were discovered to be able to stimulate the growth of pancreatic ductal adenocarcinoma (PDAC) (74). *Gamma-proteobacteria* was proposed to translocate from gut to pancreatic tumors, where they could metabolize the active form of gemcitabine, one of the most important drugs in treating PDAC, thus decreasing the chemotherapeutic effects (75). Liver is also adjacent to gut, but there is no direct duct that connects gut with liver. So, it is really hard for gut microbiota to translocate to liver, but the metabolites and excreta can be transported through blood. Through the portal vein, various of metabolites and antigens excreted from gut microbiota can be transported to liver, which thus has significant influences on liver. For example, some secondary bile acids, lipopolysaccharide and lipoteichoic acid, can promote tumorigenesis or restrain antitumor immunity in liver (76 – 80).

Let us take a close look at the short-chain fatty acids (SCFAs), one of the fermentation metabolites of gut microbiota, that might play a role in carcinogenesis, progression of CRC. Acetate, propionate, and butyrate, the three SCFAs, are the predominant energy source of colon cells, while CRC cells have the tendency of aerobic glycolysis, using glucose as the main fuel (81, 82). However, CRC cells are more sensitive to SCFAs than normal colonocytes, because SCFAs help maintain the cellular homeostasis (83). SCFAs were recently demonstrated to help induce apoptotic cell death in CRC cells and specifically, acetate could trigger the glucose metabolism changes by the mechanism of promoting the plasma membrane re-localization of monocarboxylate transporter (MCT-1), which might also be related to the initiation and progression of CRC. While butyrate, the SCFA which might be beneficial, has been shown to be a key component of preventing the deterioration of acute lymphatic leukemia by promoting the integrity of gut barrier (84). Consequently, regulating intestinal SCFA levels was becoming a potential option for CRC treatment and prevention. Additionally, SCFA transporters had connections with lactate generation and butyrate exports, which also might promote CRC initiation and progression, and was also a candidate target for treating CRC (85).

For the chemotherapy, besides the function of stimulating CRC, *F. nucleatum* could promote chemotherapy resistance of CRC. Researchers found that in the tissue of CRC patients, who had undergone recurrence after chemotherapy, *F. nucleatum* was in more abundance. After further researching, the mechanism might be that *F. nucleatum* would target toll-like-receptor-4 (TLR-4) and myeloid differentiation factor 88 (MyD88) innate immune signals and the certain microRNAs, which subsequently drove the autophagy pathway and thus influenced CRC chemotherapeutic response (9).

Considering immunotherapy, which was a novel treating strategy for patients with malignancies that proved to be a huge success (41 – 46), the most frequently used drugs were ICIs that targeted PD-1 and CTLA-4 axis. However, only a small proportion of patients with cancer derived benefit from immune checkpoint therapy (86). Consequently, the factors determining responses and resistance of ICIs therapy were figured out and then predictive markers for immunotherapy were identified to be rather critical for providing precise therapeutic strategies for every patient. To emphasize, several studies have suggested that the variety and constitution of the gut microbiota can determine ICIs therapy response (10, 15 – 21), implicating that the gut microbiota was rather an influential determinant in antitumor immunity and that the primary resistance to ICIs could be the result of abnormal gut microbiota constitutions. Studies have shown that FMT from patients who could benefit from PD-1 therapies into germ-free mice improved PD-1 blockade effects, while nonresponsive patients failed to do so (19, 87, 88). Therefore, FMT could be used with ICIs as a novel therapy. Furthermore, high microbiota diversity, regardless of species, was indicated to improve ICIs response (18, 20) and antibiotics would inhibit the clinical efficacy of ICIs in patients with cancer in advanced stage maybe due to the elimination of gut microbiota (18). Moreover, in these studies, several specific kinds of bacteria were discovered to be interrelated to the ICIs response. Relatively, plenty of *Akkermansia muciniphila* helped enhance ICI efficacy in the presence of IL-12 in non-small cell lung cancer (NSCLC), renal cell carcinoma (RCC), and urothelial cancer (18), while *A. muciniphila* also produced inosine, which might improve ICI effects (89). *Alistipes indistinctus* also helped in NSCLC (18). *Bifidobacterium* could not only promote the antitumor effect, but also improve PD-L1 therapy, having the ability to nearly terminate the tumor growth (17). Three other independent researches on melanoma, respectively, found that abundancy of *Faecalibacterium* and other *Firmicutes* (21), *Bifidobacterium longum*, *Collinsella aerofaciens* and *Enterococcus faecium* (19), *Ruminococcaceae family* (20) could improve ICIs efficacy, but detailed mechanisms still need to be fully elucidated. Also, despite the fact that *F. nucleatum* would lead to chemotherapy resistance in CRC (9), it was proved that a high level of *F. nucleatum* correlated with better responses to PD-1 blockade in patients with CRC. And supplying *F. nucleatum* with PD-L1 blockade therapy recovered the therapeutic effects a lot by stimulating stimulator of interferon genes (STING) signaling and increasing the number of interferon-γ (IFN-γ) + / CD8 +tumor-infiltrating lymphocytes (10). In addition, besides the responsiveness, adverse events induced by ICIs also decided whether the patient could use it. For instance, *bacteroidetes phylum* might decreased the risk of encountering ICI-induced colitis (90). Last but not the least, though the effect of gut microbiota was emphasized here, it was just one factor that decided the ICI therapy response. Other factors like PD-L1 expression, the density, phenotype and diversity of tumor-infiltrating lymphocytes, transcriptomic and epigenetic signatures were also very important (86). It is significant to point out that the results talked above remained to be verified whether they were generally applicable and promising in other larger independent cohorts.

### The green cluster: role of gut microbiota in immunity, inflammation, and inflammatory bowel disease

It has already been demonstrated that the gut microbiota regulates the development of some specific types of lymphocytes, which will change the immunity and inflammation state (91). Here are some examples, Th17 cells comprise one lineage of CD4+Th cells that are pro-inflammatory, which help defend pathogens (92, 93). Unlike other lineages, including Th1 and Th2 cells, Th17 cells tend to accumulate in the gut, implying the potential regulation by intestine-intrinsic mechanisms (92, 94 – 96). The promotion of Th17 proliferation may be mainly attributed to the effect of segmented filamentous bacteria but the mechanism has not been completely clarified (92). FOXP3+Tregs also composes one lineage of CD4+Th cells, which maintain immune homeostasis and accumulate in the gut. Loss of these Tregs leads to inflammation on account of abnormal expansion of other CD4+Th cells, which greatly express commensal bacteria-specific T cell receptors (97). The development of IL-10 producing Tregs could be stimulated by *Clostridium* spp., altered *Schaedler* microbiota, and *bacteroides fragilis*, but detailed mechanisms are still needed to be explained (98 – 100). In addition, gut microbiota has close link with intestinal-specific B cells that secrete IgA (101). Commensal gut bacteria can promote the gene expression of factors to induce differentiation from lamina propria DCs and/or follicular dendritic cells (FDCs) into IgA+B cells, such as tumor necrosis factor (TNF), inducible nitric oxide synthase, B cell activating factor, and TNFSF13 (a proliferation-inducing ligand of TNF) (102, 103). IgA, in turn, enhances intestinal barrier function and helps to regulate the proper composition and function of gut microbiota. Moreover, one research found that the binding of IgA to *bacteroides thetaiotaomicron*, one kind of commensal gut microbiota, hinders innate immune responses by altering its gene expression (104).

Many bacterial components such as flagellin, lipopolysaccharides (LPS), and other endotoxins, together with the metabolites like SCFAs have significant effects on the inflammatory state (105). When the gut barrier is disrupted by diet or abnormal bacteria, LPS may dislocate and recruit macrophages, which can release a lot of cytokines to promote inflammation, and these cytokines will help specific group of T cells differentiation and alter the inflammatory state (106). In addition, LPS could bind TLR on immune cells to activate pro-inflammatory cascades, sustaining steady production of neutrophils against bacterial infections (107, 108). Flagellin helps synthesize retinoic acid to promote the differentiation of IgA-producing B cells. SCFAs can help T cells differentiation into effector or regulatory T cells on the condition of different cytokine milieus, playing an important role in regulating the colon immunity homeostasis. SCFA increased CD4+Th cell number and proportion but did not change the number of colonic Th1 and Th17 cells obviously. SCFAs could induce the Foxp3+IL-10-producing Tregs specifically to maintain intestinal immune homeostasis and regulate inflammation by controlling the proliferation of effector CD4+T cells (64). The effect of SCFAs lied on the activity of direct histone deacetylase (HDAC) inhibitor. The inhibition helped acetylate p70 S6 kinase (S6K) and helped phosphorylate ribosomal protein S6 (rS6), regulating the mTOR-S6K pathway to generate Th1, Th17, and IL-10+T cells (109). Also, it was shown that the effect of SCFAs depended on the type and concentration and, for instance, butyrate was an anti-inflammatory metabolite and could alleviate inflammation in patients in a clinical study (110, 111).

Considering the study results talked above, it indicated that regulating these factors might have clinical benefits to improve patients’ inflammation, ameliorating the therapeutic effects.

IBD develops due to the combination of environmental, microbial, immunological, and genetic effects, and all of the factors are necessary. Several experimental and clinical researches have correlated gut microbiota with initiation and progression of IBD partly due to the fact that germ-free mice did not develop IBD at all or the disease conditions were attenuated largely, but the specific species and the detailed mechanisms that contribute to IBD have not been fully unmasked by researches (108, 112). Decreased biodiversity like depletion of species such as *phyla Firmicutes*, *Bacteroides fragilis*, *Bacteroidetes* is often linked to IBD patients and an increase in *Proteobacteria* also counts for IBD, but the causal and consequential effect is not clear now (113 – 116). A reasonable mechanism is that loss of gut microbiota biodiversity may contribute to a reduction of key functions, which may be necessary for sustaining intestinal barrier integrity and for regulating the host immune system, resulting in mucosal damage to cause IBD in some susceptible persons (117, 118). The mucosal immune imbalance will exhibit a characteristic that the immune tolerance mechanism is ineffective, secreting abnormal IgG antibodies instead of IgA antibodies, fighting against gut microbiota and microbial antigens (119, 120). Genetically, several genes like NOD2, ATG16L1, and IRGM were proposed as IBD-susceptibility genes, which might be linked with failing to sense protective signaling transduced from the gut microbiota (121). Following researches are needed for various unsolved problems in this field.

### The blue cluster: interaction between gut microbiota and acute injuries plus metabolic diseases

Besides the fact that gut microbiota will significantly influence chronic diseases like IBD and cancer, gut microbiota has also been found to play a significant role in the disease progression, prognosis, and recovery of acute trauma such as acute CNS injuries, burn injuries (67), as well as metabolic diseases including obesity and diabetes. Recently, many researchers found that gut microbiota will impact the acute injuries and metabolic diseases through many mechanisms. Clarifying the unknown mechanisms and developing a new therapeutic strategy like FMT and probiotics probably will provide new insights into the treatment.

Acute CNS injuries, such as stroke, spinal cord injury (SCI), and traumatic brain injury (TBI), are usual causes of lifelong disabilities or even death. Recent researches indicated that altered composition of gut microbiota after acute CNS injury would probably break the equilibrium of the patient’s bidirectional gut-brain axis (22).

First, talking of the altered composition and metabolites of gut microbiota, Bazzocchi G found that the gut microbiota of patients who suffered from SCI was not only enriched in some pro-inflammatory and/or opportunistic pathogenic bacteria but also was lacking in SCFAs producers (122). Zeng X and Tan C observed similar phenomenon in stroke patients, especially stroke patients with high risk and comparatively worse prognosis (123, 124). SCFA level was found to be negatively related to the severity and prognosis of stroke patients in one research as well (124). To figure out the intrinsic reasons, researchers demonstrated that SCFA helps structural and functional remodeling with the help of T cells recruitment to infarcted area of brain. SCFAs also could mitigate inflammation and post-stroke neurological deficits through increasing Treg cells as well as reducing IL-17^+^ γδT cells (125, 126). Also, butyrate was found to reduce brain oxidative stress, alleviate neuronal apoptosis after stroke as well as protect gut epithelial cells to prevent stroke-induced gut-derived infection (127 – 129).

Second, pro-inflammatory responses will be triggered after acute CNS injuries in both CNS and PNS, which might cause secondary brain injury, cognitive disorders, and motor dysfunctions, leading to worse prognosis (122, 130 – 137). Patients suffering stroke and TBI are found to have a higher level of trimethylamine N-oxide (TMAO) (138 – 140), which activates various pro-inflammatory responses including NLRP3 inflammasome, MAPK, and NF-κB pathways, predicting higher severity and risk (141, 142). Gut microbiota also may contribute to this phenomenon, one research transplanted post-stroke gut microbiota to germ-free mice, discovering that it exacerbates infarct volume and functional defects with pro-inflammatory T cells like Th1, Th17, and γδT cells migration into the ischemic area of brain (130).

Finally, FMT, probiotics, and prebiotics addition can help manipulate the gut microbiota, normalizing acute CNS lesion-induced dysbiosis and triggering protective immune response to alleviate further CNS injury and facilitate functional outcomes (130, 143 – 145). However, the safety and efficacy of these treatment strategies still require further investigations before they are carried out in reality.

Burn injuries are also a common form of acute traumatic injuries, which overturn normal inflammatory conditions and may cause significant alterations in organ systems and functions. Recently, it has become obvious that gut microbiota plays a significant role in regulating the immune responses and recovery from burn, as well as contributing to significant detrimental complications like sepsis (23). The relationship between burn, gut microbiota, and immune microenvironment is really complex. To be general, it can be described by three steps. First, burn injury leads to compromised mucosal immune and increased intestinal permeability. Second, bacterial translocation happens and innate immune will be acutely activated, promoting further immune dysregulation. Finally, the immune system is further disrupted, which leads to increased dysbiosis and subsequent severe complications.

To begin with, mucosal immune is greatly disturbed after burn injury. Neutrophils, the first responders, have reduced phagocytic and bacterial killing ability (146). Moreover, the adaptive immune system, which is the most important for prevention of bacterial translocation and gut-derived infection, will also be compromised with both CD4 and CD8 T cells perturbed (147, 148). Also, burn injury will reduce epithelial cell tight junction protein expression and disrupt tight junction localization (149, 150), leading to increased gut barrier permeability. The two factors collectively result in high probability of bacterial translocation.

Then, translocating bacteria and their metabolites can be rapidly sensed by systemic innate immune cells (e.g., neutrophils and macrophages) through pathogen-associated molecular patterns (PAMPs), triggering rapid onset of acute inflammation (151). Due to hypoxia and cellular damaged components after initial burn injury, activated neutrophils are overly recruited into the GI tract and macrophages will produce copious pro-inflammatory cytokines, resulting in innate immune dysregulation and acute mucosal injury and dysbiosis (152, 153). What is more, after burn injury, the neutrophils are not only worse at eliminating infections, but also have paradoxically prolonged lifetime, leading to a followed long-term immune disruption and dysbiosis (153, 154).

Finally, the dysbiotic microbiota can further deteriorate the mucosal immune dysfunction and promote bacterial translocation, resulting in sepsis and even MODS (155, 156). Consequently, there is a positive-feedback loop between immune dysregulation, dysbiosis, and bacterial translocation after the burn injury trigger, leading to uncontrollable inflammation and septic complications.

Besides, some specific families of gut microbiota were found to be altered in severe burn. First, the originally dominated *Bacteroidaceae* and *Rimunococcaceae* families are significantly decreased while the *Enterobacteriaceae* family, which contains pathogenic *E.coli*, is observed to have an increase in the abundance (157). Second, the altered abnormal composition of gut microbiota will further deteriorate the burn injuries. *Bacteroidaceae* and *Rimunococcaceae* families are the main producers of SCFAs, and as talked above, SCFAs can serve as a source of energy, modulate immune conditions, and promote gut barrier integrity (158 – 160). Therefore, decreased concentration of SCFAs further disturbs the gut function as well as immune microenvironment and then exacerbates the burn injuries in a comparatively long time (157, 161). Worse still, the increasing amount of *E.coli* may promote the happening of complications of burn injury (162). In a conclusion, breaking these vicious cycles might be an important aspect of treating burn injuries, which rather deserves following researches in the future.

Gut microbiota affects other acute injuries like severe acute pancreatitis-associated lung injury (PALI), acute liver injury, and acute radiation syndrome as well. For instance, in PALI, SCFAs will also participate in the fight, which means the inhibition of pathogens, enhancement of gut barrier and anti-inflammation (163). In acute radiation syndrome, radioprotective microbes including *Lachnospiraceae* and *Enterococcaceae* together with metabolites such as propionate and tryptophan metabolites are associated with better post-radiation restoration (24).

Consequently, figuring out the role of gut microbiota in acute trauma treatment would probably be an exciting frontier in helping improve the prognosis and clinical outcomes.

The prevalence of metabolic diseases is rapidly increasing, and it was observed that gut microbiota might influence the metabolic health (164). And its relationship with obesity, type 2 diabetes mellitus (T2DM), cardio-metabolic diseases (CMD), and non-alcoholic fatty liver disease (NAFLD) was more focused in our discussion. First of all, it was found that the composition and function of the gut microbiota shared many similarities in common metabolic diseases, and high biodiversity characterizes healthy gut microbial communities (165). Obesity, T2DM, and other insulin-resistant diseases feature chronic and unresolved tissue inflammation (166). It was found that the butyrate producers including the *Oscillospira* spp. and *Methanobrevibacter smithii* were more associated with lean people (167, 168). Besides, prediabetes patients tended to have a low abundance of butyrate-producing bacteria, while exhibiting an increase in pro-inflammatory taxa (169, 170). Gut microbial dysbiosis was also linked to CMD, demonstrating a more inflammatory but less fermentative microbiome, with increasing *E. coli*, *Klebsiella* spp., and *Enterobacter aerogenes* (117), but decreasing *Bacteroides* spp. and anti-inflammatory *Faecalibacterium prausnitzii* (171). In addition, the gut microbiota of NAFLD patients was enriched in ethanol-producing bacteria (172), and ethanol could activate nuclear factor-κB (NF-κB) signaling pathways to induce inflammation (173). Finally, it should be emphasized that together with many other regulatory mechanisms, the cause-and-effect investigations are needed in the future.

**Fig 9:**
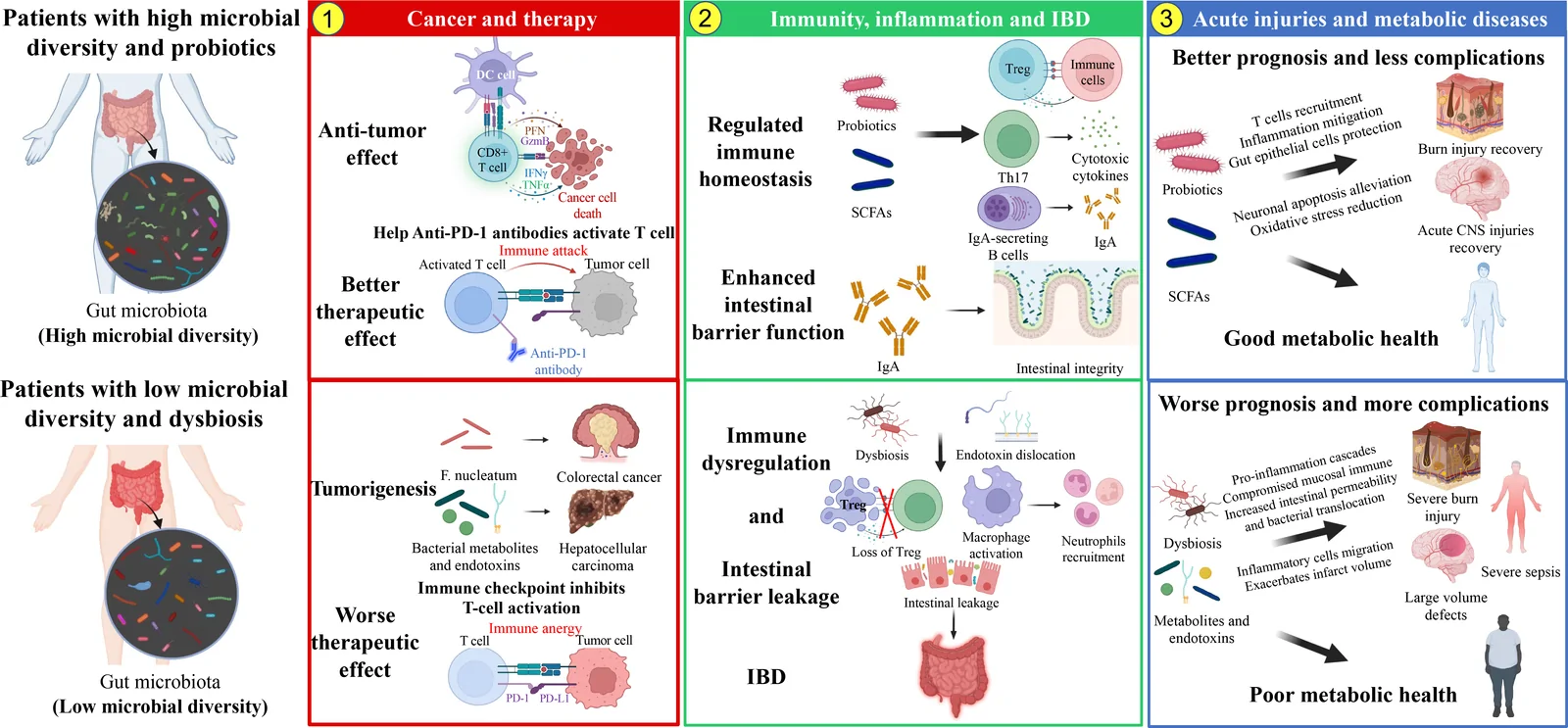
Three major clusters in our discussion section. Red cluster concerning gut microbiota with cancer and therapy. Green cluster discussed the relationship between gut microbiota and immunity, inflammation plus IBD. Blue cluster talked about the topics of acute injuries and metabolic diseases.

In the end, we summarized the discussion section in Fig. 9, which could intuitively demonstrate the contents talked above.

### Limitation

This is the latest bibliometric analysis of documents in the gut microbiota and immune microenvironment field, which summarizes the publications from 1976 to 2022. However, despite achieving several meaningful and visualized findings, flaws inevitably exist due to the data acquisition method. The documents were all retrieved only from the WOS Core Collection database, and thus the documents relevant to the field in other sources are not available. In addition, for the new articles that were published after our retrieval date could not be involved in our analysis. In further research, we can collect the documents from multiple data sources to acquire more comprehensive analysis and a broad view of this field.

## Data Availability

The datasets generated and/or analyzed during the current study are available on the Web of Science (WOS, http://www.webofknowledge.com). Due to the frequent renewal of WOS database, the retrieval results might vary at different retrieval time, so we uploaded our retrieval data in the supplementary material (called "download 2023.txt").

## SUPPLEMENTAL MATERIAL

download 2023.txt spectrum.00135-23-s0001.txt Our retrieval data generated and analyzed during the study from the Web of Science (WOS).

Supplemental figures spectrum.00135-23-s0002.txt Fig. S1 to S9.

ASM does not own the copyrights to Supplemental Material that may be linked to, or accessed through, an article. The authors have granted ASM a non-exclusive, world-wide license to publish the Supplemental Material files. Please contact the corresponding author directly for reuse.
